# Prevalence of delay in seeking tuberculosis care and the health care seeking behaviour profile of tuberculous patients in a rural district of KwaZulu Natal, South Africa

**DOI:** 10.11604/pamj.2021.39.27.26717

**Published:** 2021-05-11

**Authors:** Linda Chiposi, Lindiwe Priscilla Cele, Mathildah Mokgatle

**Affiliations:** 1 Epidemiology and Biostatistics Unit, Department of Public Health, Sefako Makgatho Health Sciences University, Ga-Rankuwa, South Africa

**Keywords:** Primary health care, South Africa, prevalence, cross sectional, pharmacy

## Abstract

**Introduction:**

patient delay in seeking TB (tuberculosis) care is reported as one of the major hurdles undermining the efforts of controlling TB by many TB control programmes of the world. The main aim of this study was to determine the prevalence of this phenomenon and to profile the TB patients that delayed seeking TB care in a rural area of KwaZulu Natal province of South Africa.

**Methods:**

this was a cross-sectional study, conducted among 200 TB patients attending primary health care facilities in Ugu District. Patient data were collected by a self-administered questionnaire, entered into an Excel file and imported into the EpiInfo 7 statistical software for analysis. Frequency tables were used to display the data and the p value was used for statistical significance.

**Results:**

about 40% of the participants delayed seeking TB care in this study, and these were mostly individuals who were married, the employed and those who walked to the clinic. Delay was also prevalent among those that self-medicated, bought medication from the pharmacy and sought TB care from a private doctor. The reasons included the great distances, long queues waiting at the facilities, and not feeling ill.

**Conclusion:**

the 4 weeks cut-off in seeking TB care in this study far exceeds the recommended 2 weeks. This study recommends periodic active TB case finding and active engagement between the public and the private health sectors.

## Introduction

Tuberculosis (TB) remains one of the top ten (10) causes of death globally, with 10 million people reported to have fallen ill from the disease worldwide in 2018. An estimated 1.7 billion people are said to have been infected with latent TB in the same year, with over 25% of TB deaths reported to have occurred in the African region [1,2]. In South Africa, not only is TB a major health problem with 520 per 100,000 new cases reported to have occurred in 2018, but TB-drug resistance and HIV co-infection rates are also said to be high. The country ranks fifth among the countries with the highest rifampicin resistance (RRTB) with an estimated 59% of TB patients with known HIV status reported to be HIV-positive [1,3]. KwaZulu Natal (KZN) is one of South Africa´s provinces that is heavily burdened with TB. The first cases of extensively drug-resistant TB (XDRTB) were detected in KZN´s uMsinga sub-district, in the Tugela Ferry, in 2006 [4]. The Ugu district where this study was conducted has TB as the leading cause of death especially among the younger (5-14 year-old) and the older (15-64 year-old) people. This district also had the greatest number of reported TB cases in 2014 and the highest TB-HIV co-infection rate of 70% [5,6].

In responding to the TB epidemic, the country has adopted the WHO-endorsed rapid molecular method of TB diagnosis, GeneXpert MTB/RIF (Xpert), which replaces smear microscopy as the first-line test for the diagnosis of both drug-susceptible (DS) and rifampicin-resistant (RR) TB in 2012, and the line probe Assay (LPA). Although the Xpert presents some limitations, which include that of missing mutations associated with the rifampicin resistance-determining region (RRDR) of the rpoB gene [7,8], its introduction has, however, seen a massive increase in the number of TB patients initiated on anti-TB drugs in South Africa from 5,083 in 2009 to 12,640 in 2015 [9]. The country has also updated its guidelines on the management of RRTB in line with the World Health Organisation´s (WHO) recommendation on all-oral regimens for the treatment of RRTB [10].

One of the major hurdles that has been reported by many TB control programmes worldwide is patient delay in seeking TB care after experiencing the signs and symptoms of TB. This undermines the efforts that are crucial for the effective control of TB, as it means that TB suspects will not be diagnosed early and started on effective anti-TB treatment immediately, as recommended by the WHO. It is reported that individuals with active TB can infect between 5 and 15 close contacts annually, and it is also said that without proper TB treatment, 45% of those who are HIV-negative and all of those who are HIV-positive will die, on average [11]. According to the South African TB guideline, any person that is diagnosed with drug susceptible TB (DSTB) or drug resistant TB (DRTB) should be started on anti-TB drugs within 2 days and 5 days respectively [12]. Several studies have reported patient delays of between 30 and 180 days before seeking TB care. In a study that was conducted among new TB patients between 2013 and 2014 in a municipality of Ghana, the median patient delay period was 59 days. In another that was conducted in Northwest Ethiopia, the mean and the median delays were 45 and 30 days respectively [13,14].

Other studies include one that was done among PTB pulmonary TB cases in a government specialist hospital in Ibadan, Nigeria, where the median delay was 60 days (range 3-180 days) [15]. Another was conducted in the North West zone of Tigrai region of Ethiopia, where the delay was 30 days (IQR 21-60 days) [16]. In one study that was conducted in a rural Northern Province of South Africa, the median patient delay was 4 weeks and this is said to have translated to 7.2/100 person weeks of coughing in the community [17]. The influences of gender, the level of education, employment status, age and place of residence have been reported by some studies [15,18]. Others report gender and marital status as being associated with delay in seeking TB care [19]. Addressing patient delay in seeking TB care should be a priority of every TB control programme, more especially in countries in sub-Saharan Africa (SSA), a region where several countries rank among the highest burden countries globally, with high rates of TB incidence [1]. The objective of this study was to determine the prevalence of delay in seeking TB care and to characterize the profile of TB patients who delayed in seeking TB care at primary health care facilities at Ugu district of KwaZulu Natal.

## Methods

**Study design:** this study utilized a cross sectional study design.

**Study setting and study area:** the study setting were 5 primary health care facilities in the Ugu district. The district is situated on the South Coast of KZN, and has four (4) local municipalities, namely Umdoni, Umzumbe, Ray Nkonyeni and uMuziwabantu. The total population was approximately 753,336 inhabitants in 2016, with the majority (76%) living in rural areas [6]. The majority (90%) of the people are Africans and the population is relatively young; children up to age 14 comprise 38% with individuals between the ages of 15-64 years old making up 58% and the remaining 4% being the elderly, people over 65 years of age [20].

**Study population:** the study population were the DSTB patients who were attending the selected health facilities of Ugu district between 15^th^ May and 15^th^ September 2017. The study sample were DSTB patients that had been initiated on first-line anti-TB drugs for not more than 3 months.

**Sample size determination:** for sample size determination, the study used the Open-Epi version 3 open source calculator. With a total 2,039 of TB patients that had been initiated on anti-TB drugs in 2016 and assuming a hypothesized outcome factor (p) of 50% in the population, and setting a confidence limit of 5%, the sample size was determined to be 324.

**Sampling technique:** this study recruited participants from among all the TB patients that were attending the health facilities between 15^th^ May and 15^th^ September of 2017 if they met the inclusion criteria.

**Inclusion and exclusion criteria:** the study enrolled all the TB patients who were 18 years and above and that had been initiated on first-line anti-TB drugs for not more than 3 months. Very ill patients and those who were mentally ill were excluded from participating which resulted in a final sample size of 200 participants.

**Data collection method:** participant information was collected using a self-administered questionnaire. The variables included participant sociodemographic characteristics (age, gender, marital status, level of education, employment status, residential setting - urban, rural, informal, homeless, displaced - number of rooms in the home, the number of occupants) and the mode of transport they used to get to the clinic; information on patient delay, including the reasons for the delay; the first action taken to get treatment, and the health-seeking behaviour.

**Data analysis:** the data were entered into an Excel spreadsheet and imported into the Epi Info 7 statistical software package for analysis. The mean or the median and the corresponding standard deviations (SD) and interquartile ranges (IQR) were used to present continuous variables. The continuous variables age and number of rooms were recoded and converted into categorical data for further analysis. These variables were presented as proportions and percentages, along with the other categorical data, such as the reasons for the delay in seeking TB care.

**Ethical consideration:** ethical clearance was obtained from the Ethics and Research Committee of Sefako Makgatho Health Sciences University (SMUREC/H/199-2015: PG). Permission to conduct the research at the Ugu District Health facilities was obtained from the department of health-Ugu health district office. All prospective participants were requested to provide individual informed consent prior to the interview.

## Results

**Sociodemographic characters:** a total of 200 TB patients were enrolled into the study. Of the 200 TB patients, 185 (93%) were eventually enrolled, as 15 were excluded due to issues of recall on the period of delay in seeking TB care after onset of symptoms. The median age of the remaining 185 participants was 35 years (IQR=15). The majority (41%) were in the 29-39 year age group, 82% were single, 56% were male, 62% reported having a high-school education, 57% were unemployed, 73% were living in a rural setting, 48% were living in a house with between 1 and 3 rooms and 51% using a taxi as their mode of transportation.

**Delay in seeking TB care:** in this study, a participant was considered to have delayed seeking TB care if the time she/he took to seek TB care from a health facility after experiencing TB symptoms, exceeded 4 weeks (1 month). Table 1 shows that 40% (74/185) of the participants delayed seeking TB care. Among those that delayed, most (45%) were married participants, 41% were male, 57% were older than 61 years, 43% had high-school education, 50% were living in informal settlement, 41% were unemployed, 47% were living in houses with more than 6 rooms, and 43% of them walked to the clinic, not having another means of transportation.

**Table 1:** sociodemographic characteristics of participants stratified by delay status, N=185

	Delay in seeking TB care		
	Yes (n=74)	No (n=111)	Total (%)
**Gender**			
Female	32 (39)	49 (61)	81 (44)
Male	42 (41)	61 (59)	103(56)
**Age**			
Median (IQR)	35(15)		
**Age group**			
18-28	15 (36)	27 (64)	42 (23)
29-39	33 (43)	43 (57)	76 (41)
40-50	14 (34)	27 (66)	41(22)
51-61	8 (42)	11 (58)	19 (10)
>61	4 (57)	3 (43)	7 (4)
**Marital status**			
Married	15 (45)	18 (55)	33 (18)
Single	59 (39)	93 (61)	152 (82)
**Level of education**			
Tertiary	5 (31)	11 (69)	16 (9)
High school	46 (40)	68 (60)	114 (62)
Primary	22 (43)	29 (57)	51 (27)
No school	1 (33)	2 (67)	3(2)
**Residence setting**			
Informal settlement	10 (50)	10 (50)	20 (11)
Rural	52 (39)	83 (61)	135 (73)
Urban	12 (40)	18 (60)	30 (16)
**Employment status**			
Employed	31 (39)	49 (61)	80 (43)
unemployed	43 (41)	62 (59)	105 (57)
**Number of rooms in the household**			
Mean (SD)	4 (2)		
1-3	32 (36)	56 (64)	88 (48)
4-6	34 (43)	45 (57)	79 (43)
>6	8 (47)	9 (53)	17 (9)
**Mode of transport**			
Bus	1 (33)	2 (67)	3(2)
Car	5 (33)	10 (67)	15 (8)
Taxi	37 (39)	58 (61)	95 (51)
Walk	31 (43)	41 (57)	72(39)

**Reasons for the delay in seeking TB care:** almost half (47%) of the participants indicated that they had not sought TB care early because they had not felt so bad and these were mostly female (53%). Others stated that the health facility was too far away (27%) and of these, the majority were male (65%). About 19% said they were afraid of the long waiting times and these were mostly male (64%). A few (3%) reported that they did not have transport money, while another 3% stated that they could not take the time off work and 1% said they were fearful of the nurses (Table 2).

**Table 2:** reasons for the delay in seeking TB care stratified by gender, n=74

	Frequency (%)		
	Total (n=74)	Female (n=32)	Male (n=42)
Health facility too far	20 (27)	7 (35)	13 (65)
I am afraid of long waiting time	14 (19)	5 (36)	9 (64)
I did not feel too ill	34 (47)	18 (53)	16 (47)
I had no money for transport	2 (3)	1 (50)	1 50)
I fear nurses	1 (1)	0 (0)	1 (100)
I could not take time-off work	2 (3)	0 (0)	2 (100)
No reason	1(1)	1 (3)	0 (0)

**Health-care seeking patterns:** for the first action for seeking TB care after symptom onset, the majority (69%) reported that they saw a nurse, (24%) saw a private doctor, (7%) bought medication from the pharmacy and 3% reported self-medicating. The prevalence of delay in seeking TB care was high among those that reported self-medicating (80%), buying medication from a pharmacy (46%) and seeing a private doctor (46%). The reasons that were given for the choice of the first action include 46% who stated that a friend or a family member advised them, (42%) who indicated that the service was free and affordable, (40%) who were sure that they would get better faster and 35% who reported that the clinic was nearby and that it was quick with no time wasted waiting for help. The main reported TB symptoms were cough (80%) and loss of weight (80%). More than half (64%) of the participants were HIV co-infected and of these, 37% had delayed in seeking TB care (Table 3).

**Table 3:** health care seeking patterns and the clinical history, stratified by delay status, N=185

	Delay in seeking TB care			
	Yes (n=74)	No (n=111)	Total (%)	P value
**First action**				
I saw a nurse	46 (36)	82 (64)	128 (69)	0.169
I saw a private doctor	18(46)	21 (54)	39 (21)	
I self-medicated	4 (80)	1 (20)	5 (3)	
Bought medicine from the pharmacy	6 (46)	7 (54)	13 (7)	
**Reason for the first action**				
It was nearby	32 (35)	60 (65)	92 (50)	0.183
I was sure I will get better fast	52 (40)	79 (60)	132 (71)	0.953
It was quick, no time wasted waiting for help	28 (35)	51 (65)	79 (43)	0.320
I was advised by a friend or family member	38 (46)	45 (54)	83 (45)	0.138
It was free or affordable for me	33 (42)	46 (58)	79 (43)	0.651
**TB symptoms experienced**				
Chest pain	54 (73)	80 (72)	134 (72)	0.893
Cough	59 (80)	94 (84)	153 (83)	0.480
Fever	49 (66)	68 (61)	117 (63)	0.033*
Loss of weight	59 (80)	92 (83)	151 (82)	0.587
Night sweat	54 (73)	80 (72)	134 (72)	0.893
**HIV status**				
Positive	43 (37)	74 (63)	117 (64)	0.339
Negative	29 (44)	37 (56)	66 (36)	

## Discussion

The results of this study indicate that a high proportion (40%) of the participants delayed seeking TB care. This is a disturbing finding, as the cut-off delay of 4 weeks far exceeds the WHO recommended 2-weeks for initiating treatment after suspicion. These symptomatic and untreated TB suspects represent a source of contagion that continues to transmit TB among close contacts in households and communities. Studies done in Eastern Ethiopia and Zimbabwe also found delays among 46% and 48% of the participants respectively [19,21]. The high incidence of delay in seeking TB care among married participants (45%) could be because this particular group of individuals is supposedly inundated with responsibilities compared to their unmarried counterparts. A study that was conducted in Northern Central Ethiopia found shorter delays among widowed and divorced participants [22]. Other studies have reported much higher proportions (67%) of married people that delayed seeking TB care [23].

This study also found that the delay in seeking TB care was higher among participants that walked to the clinic than among those that used other modes of transportation. This could be explained by the long distances that were indicated by 27% of the participants, who stated that they delayed seeking TB care because the health facilities were too far away. Studies that were conducted in Ethiopia and Nigeria also found a high prevalence of delay among participants that did not have access to modern or traditional means of transportation [15,24,25]. A delay in seeking TB care was prevalent among participants older than 61 years. Similar results were observed in studies that were conducted in Northern Central Ethiopia and Northwest Ethiopia, which found delay to be high among participants over the age of 55 years [22,26]. This particular group of people has special physical and financial needs that might explain the delay in seeking TB care.

This study also examined the reasons for the delay in seeking TB care as well as the health-care seeking pattern among the participants. Almost half (47%) of the participants stated that they had not sought TB care because they had not been feeling ill. This is indicative of the low TB risk perception among the participants and to our knowledge, no study has reported a similar finding. Long queues in which one has to wait were another reason given by 19% of the participants. Another study found that 7% of its participants reported long waiting times as the reason for delaying seeking TB care [23]. The delay prevalent among the participants who had sought TB care from a private doctor, bought medication from the pharmacy or self-medicated has also been reported by other studies [26]. This could be because of the long queues that were given as a reason for the delay in seeking TB care by some participants. The delay among participants that reported coughing and a loss of weight has also been reported in some studies [27]. This could be due to the stigma emanating from the association of these symptoms with other illnesses or diseases.

**Limitations:** this study assessed only patient-level factors influencing the delay in seeking TB care. The failure to reach statistical significance in the differences observed in this study could be due to the small sample size.

## Conclusion

The proportion of participants that delayed seeking TB care in this study was large. The long queues in the health facilities, as reported by some participants, are an indication of the system´s failure to meet the demand for the service and this needs to be addressed. A study that was conducted to assess clinic flow among STI, HIV and TB patients in Durban found an overall 6 hours and 56 minutes time from arrival to exit for TB patients. This included the time between testing and treatment initiation [28]. This could be the cause of some of the participants choosing to seek TB care elsewhere.

Periodic active case finding and mass education among communities, especially in places of high prevalence, should be on the agenda of any TB control programme. This would not only help find cases but would also increase awareness and TB risk perception among the suspects, and families would be empowered to identify TB suspects among their family members. Although only about 16.7% of the South African population utilises a private health service [9], this minority constitutes a significant proportion of the population, considering the TB disease and how it is transmitted. Attention should focus on private provider knowledge, skill, and the availability of treatment and the diagnostics. There needs to be an active engagement between the public and the private health sectors especially in regard to training in TB diagnostic algorithms and interpretation, as these private patients will ultimately seek TB care from the public health facilities once their medical funds become exhausted.

### What is known about this topic

- Long queues in public health facilities cause patients to seek health care elsewhere but ultimately, they seek TB care from the public health care facilities;
- A minority of patients seek health care from private doctors and among these are TB suspects who seek TB care from the public health sector only when their medical aid scheme funds have been exhausted.

### What this study adds

- The low TB risk perception among TB suspects needs to be addressed;
- The public and private health care sectors need to engage actively to address the delay among TB suspects that seek TB care from private doctors first and then seek TB care from public health care facilities only later.
